# Individualized regulation of inflatable laryngeal mask airway cuff pressure reduces postoperative pharyngolaryngeal pain in elderly patients: a randomized controlled trial

**DOI:** 10.3389/fmed.2026.1803147

**Published:** 2026-05-15

**Authors:** Xuanqi Yang, Sixu Lai, Su Min, Wenjie Cheng, Dayuan Wei, Chengye Ren

**Affiliations:** Department of Anesthesiology, The First Affiliated Hospital of Chongqing Medical University, Chongqing, China

**Keywords:** elderly patients, inflatable laryngeal mask airway, pharyngolaryngeal pain, pressure individualized regulation, randomized controlled trial

## Abstract

**Background:**

Inflatable laryngeal mask airway (ILMA) cuff pressure lacks routine monitoring and standardized safe thresholds, with clinical practice often referencing manufacturer specifications. Empirical inflation in clinical practice often leads to overinflation and increased pharyngolaryngeal complications. This study established an individualized ILMA cuff pressure regulation strategy based on physiological characteristics and sealing principles, aiming to compare its effect on postoperative pharyngolaryngeal pain in elderly patients with empirical inflation.

**Methods:**

This single-center, double-blind randomized controlled trial comprised 78 elderly patients undergoing general anesthesia with ILMA. Patients were randomly assigned to either the empirical inflation (EI) group or the individualized regulation and real-time monitoring (RM) group. In group EI, the cuff was inflated empirically. In group RM, individualized regulation and real-time monitoring were applied: cuff pressure was adjusted to maintain ≥ peak airway pressure, with the oropharyngeal leak pressure (OLP) ≥ 25 cmH_2_O. The primary outcome was the incidence of pharyngolaryngeal pain within 48 h after ILMA removal. The secondary outcomes included other pharyngolaryngeal complications (e.g., supraglottic mucosal injury graded by fiberoptic bronchoscopy), 7-day pulmonary complications assessed by the Postoperative Pulmonary Complications (PPCs) scale and other prespecified secondary outcomes.

**Results:**

78 elderly patients were randomized, and 74 completed the study. Cuff pressure was significantly higher in group EI than in group RM (*p <* 0.001), with no between-group differences in ILMA positioning accuracy or ventilation sealing performance (*p >* 0.05). Group RM had a lower incidence of pharyngolaryngeal pain (7.7% vs. 35.9%, *p* = 0.003) and mucosal injury (0% vs. 15.4%, *p* = 0.011) compared with group EI. The reduction in postoperative pain was mainly observed during the early postoperative period.

**Conclusion:**

In elderly patients undergoing ILMA-assisted general anesthesia, empirical inflation may result in unnecessarily high cuff pressure. Individualized cuff pressure regulation can reduce postoperative pharyngolaryngeal pain and mucosal injury without compromising ventilation seal integrity, and may represent a feasible strategy to optimize ILMA management in elderly patients.

**Clinical trial registration:**

ClinicalTrials.gov, identifier NCT06954857.

## 1 Introduction

As a key supraglottic airway management tool, the laryngeal mask airway (LMA) is widely used in general anesthesia and emergency settings due to its advantages of easy insertion and minimal hemodynamic impact. However, postoperative complications, including pharyngolaryngeal pain, hoarseness, dysphagia, and nerve injury, may still occur after LMA use. Among these, pharyngolaryngeal pain is the most frequent and distressing complication. Although often regarded by anesthesiologists as a mild and self-limiting issue, it remains one of the primary causes of anesthesia-related postoperative discomfort, directly affecting patients’ perioperative comfort and rehabilitation outcomes (1).

For inflatable laryngeal mask airways (ILMAs), overinflation of the cuff can compress the pharyngolaryngeal mucosa, causing local tissue ischemia, edema, and even nerve injury (2). The risk of complications increases progressively with higher cuff pressure (3). In contrast, continuous monitoring and maintenance of ILMA cuff pressure within a low, safe range have been shown to reduce the incidence of complications, for example pharyngolaryngeal pain (4). Studies have demonstrated that when cuff pressure exceeds 60 cmH_2_O, the risk of pharyngolaryngeal complications increases by 50% (5). Restricting the cuff pressure of ILMA to 44 mmHg (60 cmH_2_O) in clinical anesthesia practice can help reduce throat discomfort and lower the incidence of pharyngolaryngeal complications (6, 7). Nevertheless, there is still no unified and well-defined clinical standard for the specific safe range of ILMA cuff pressure.

The physiological basis for defining a “safe range” originates from the mechanical principle of ILMA seal: the cuff pressure must continuously counteract the airway pressure generated during positive-pressure ventilation to prevent air leakage. Therefore, the lower safety limit of cuff pressure is inherently defined by the patient’s peak airway pressure. Based on clinical practice, for the second-generation ILMA, an oropharyngeal leak pressure (OLP) of at least 25 cmH_2_O is required to ensure reliable sealing performance and meet ventilation requirements in most cases. However, in routine clinical practice, cuff pressure monitoring for ILMAs is not universally implemented, and the optimal approach to regulating ILMA cuff pressure within a relatively safe range remains unclear. Cuff inflation of ILMAs still relies predominantly on tactile-based empirical judgment (8). This subjective method has poor accuracy and tends to result in cuff pressure exceeding the safe range, highlighting the necessity of objective monitoring and precise regulation of ILMA cuff pressure.

With the increasingly prominent aging trend of the surgical patient population, this issue has become particularly critical. Elderly individuals are often accompanied by physiological changes such as pharyngolaryngeal muscle atrophy and mucosal tissue degeneration, and their tolerance to ILMA cuff pressure may be more limited (9, 10). However, most existing studies on ILMA cuff pressure management have focused on adult populations or children without stratification by age, and there remains a paucity of evidence regarding individualized cuff pressure strategies tailored to the physiological characteristics of elderly patients. Therefore, within the ERAS framework, it is clinically important to investigate whether continuous monitoring and individualized regulation of ILMA cuff pressure can reduce postoperative pharyngolaryngeal complications in elderly patients.

Accordingly, this study aims to investigate the effect of continuous monitoring and individualized regulation of ILMA cuff pressure in comparison with the conventional empirical inflation method on pharyngolaryngeal pain in elderly patients via a randomized controlled trial (RCT), so as to provide evidence-based support for optimizing clinical practice.

## 2 Methods

### 2.1 Study design

This study was a prospective, single-center, randomized controlled trial with blinded patients, postoperative outcome assessors, and data analysts. The study protocol was approved by the Ethics Committee of the hospital (2025-218-01) and registered on ClinicalTrials.gov (NCT06954857). The trial was conducted in strict accordance with the Declaration of Helsinki and reported following the Consolidated Standards of Reporting Trials (CONSORT) guidelines. All enrolled patients provided written informed consent.

### 2.2 Inclusion and exclusion criteria

Elderly patients undergoing general anesthesia with an ILMA were enrolled in the present RCT at the hospital, with recruitment conducted from May 2025 to September 2025.

The inclusion criteria were as follows: (1) Aged ≥ 60 years; (2) Non-cardiac, non-thoracic, and non-head and neck surgery; (3) Non-laparoscopic surgery; (4) Elective surgery; (5) Surgical position: supine position; (6) American Society of Anesthesiologists (ASA) physical status classification I-III;(7) New York Heart Association (NYHA) cardiac function classification I-II; (8) Expected surgical duration ≥30 min and ≤ 2 h; and (9) Body mass index (BMI) 18.5–30.0 kg/m^2^.

The exclusion criteria were as follows: (1) Preoperative predictable difficult airways, such as trismus, limited neck mobility, and other related conditions; (2) Preoperative pharyngeal and laryngeal complications including sore throat, hoarseness, blood-tinged sputum, and dysphagia; (3) Preexisting conditions such as loose teeth, laryngeal obstruction, laryngeal edema, acute airway inflammation, and gastrointestinal bleeding; (4) Comorbidities of respiratory diseases like chronic obstructive pulmonary disease (COPD) and asthma; (5) Allergies to ILMA materials (e.g., silicone, polyvinylchloride [PVC]); (6) Inability to cooperate with the study for any reason; (7) Participation in other clinical trials within 3 months prior to enrollment in this study; and (8) Any other circumstances deemed inappropriate for inclusion by the investigators. Although the Mallampati classification is a well-recognized predictor of difficult airway, it mainly reflects the risk of difficult tracheal intubation. In this study, the exclusion criterion of preoperative predictable difficult airways was applied to identify patients at risk of difficult LMA insertion. Mallampati grade alone was not used as an independent exclusion criterion.

### 2.3 Randomization and blinding

Patients were randomly assigned in a 1:1 ratio to the group EI (Empirical inflation) or the group RM (Regulated and monitored ILMA), with 39 patients allocated to each group, by computer-generated random numbers. All patients and the researchers who responsible for postoperative follow-up and data processing were blinded to group allocation. Owing to the procedural differences between group RM and group EI, the anesthesiologists performing ILMA insertion and cuff pressure adjustment were not blinded. A predefined unblinding protocol was strictly implemented to ensure trial integrity and participant safety. Emergency unblinding was only permitted in the event of life-threatening adverse events (e.g., severe laryngospasm, refractory hypotension, or suspected aspiration) that necessitated immediate confirmation of group allocation for optimal clinical management.

### 2.4 Perioperative management

Prior to surgery, participants were required to fast for 8 h and abstain from oral fluids for 6 h. Upon entering the operating room, standard monitoring of electrocardiogram, pulse oximetry, and non-invasive blood pressure were established. A peripheral venous access was opened, and lactated Ringer’s solution (Sichuan Medco Pharmaceutical Co., Ltd., Sichuan, China) was infused at a rate of 6 mL/kg/h.

General anesthesia was induced and maintained with a combined intravenous-inhalational regimen in accordance with the standard operating protocol of the operating room. Induction involved weight-based doses: midazolam 0.1 mg/kg (Jiangsu Nhwa Pharmaceutical Co., Ltd., Jiangsu, China), sufentanil 0.4 μg/kg (Yichang Humanwell Pharmaceutical Co., Ltd., Yichang, China), dexamethasone 10 mg (Chenxin Pharmaceutical Co., Ltd., Shandong, China), ondansetron 4 mg (Fu’an Pharmaceutical Group Ningbo Tianheng Pharmaceutical Co., Ltd. Zhejiang, China), etomidate 0.2 mg/kg (Jiangsu Nhwa Pharmaceutical Co., Ltd., Jiangsu, China), and vecuronium bromide 0.1 mg/kg (Zhejiang Xianju Pharmaceutical Co., Ltd., Zhejiang, China). After mask preoxygenation with controlled ventilation, ILMA was inserted when a bispectral index (BIS) of 40–60 (Guizhou Enkeda Medical Technology Co., Ltd., Guizhou, China), loss of consciousness, jaw relaxation, and hemodynamic stability were achieved. Maintenance included intravenous remifentanil 6–8 μg/kg/h (Yichang Humanwell Pharmaceutical Co., Ltd., Yichang, China), propofol 1.6 mg/kg/h (Aspen Pharma Trading Limited, Ireland), and inhaled sevoflurane 0.7 minimum alveolar concentration (MAC) (Maruishi Pharmaceutical Co., Ltd., Imazu Plant, Japan). During the operation, the dosage of anesthetic agents was adjusted appropriately based on BIS monitoring (target range: 40–60) and clinical requirements.

A disposable intubating ILMA (Zhejiang Shuguang Technology Co., Ltd., Zhejiang, China) was uniformly used in this study. The size of the ILMA was selected based on the standard body weight calculated by the Broca formula: Size 3.0 for 30–50 kg, size 4.0 for 50–70 kg, size 5.0 for 70–100 kg. Pre-insertion checks included device integrity, cuff airtightness (via cuff pressure monitor) (Zhejiang Shuguang Technology Co., Ltd., Zhejiang, China), full deflation, and water-soluble lubrication. Volume-controlled ventilation was set at 6–8 mL/kg tidal volume and 12–14 breaths/min, with effective ventilation confirmed by symmetrical breath sounds, <50 mL tidal volume discrepancy, stable SpO₂, and a rectangular P_ET_CO₂ waveform. Supraglottic mucosal condition was re-evaluated for all patients at ILMA removal. All intraoperative procedures were performed by anesthesiologists with at least 5 years of experience and not involved in postoperative outcome assessment.

At the end of surgery, patients were transferred to the post-anesthesia care unit (PACU) in the supine position with the ILMA in place. During movement and transfer, stable head and neck immobilization was maintained, continuous oxygen supply was provided, and ventilation status was monitored. The ILMA was removed only after the recovery of consciousness, spontaneous breathing, swallowing reflex, and airway protective reflexes. Patients voluntarily chose whether to use a patient-controlled analgesia (PCA) pump postoperatively. The PCA pump formulation consisted of sufentanil citrate injection 100 μg, flurbiprofen axetil 100 mg, ondansetron hydrochloride injection 8 mg, and normal saline injection 83 mL. The pump parameters were set as follows: total volume 100 mL, loading dose 2 mL, continuous infusion rate 1.5 mL/h, patient-controlled bolus dose 2 mL, lockout time 15 min, and maximum hourly dose 13 mL/h. Relevant indicators were recorded, and postoperative follow-up was performed at the prespecified time points.

### 2.5 Group interventions

In the EI group, anesthesiologists inflated the ILMA cuff empirically using a 20 mL syringe according to routine clinical practice, with the goal of achieving effective sealing and adequate ventilation. The cuff pressure was then measured and recorded using a cuff pressure monitor; however, the measured value was not disclosed to the anesthesiologist, and no further adjustment was made. This design was intended to reflect conventional empirical management while allowing objective observation of cuff pressure.

In group RM, the ILMA cuff was first inflated to 40cmH_2_O using a pressure monitor, followed by continuous monitoring. This initial pressure was chosen based on previous studies (11), pre-experimental data and clinical observation, which indicated that it was sufficiently high to ensure an OLP ≥ 25 cmH_2_O in the vast majority of pilot cases, thereby providing a safe and consistent starting point for downward regulation. Then the OLP was measured. OLP measurement method (12): manual positive-pressure ventilation mode, closed APL valve, 3 L/min oxygen flow, with plateau pressure at audible mouth leakage defined as OLP.

**Figure 1:**
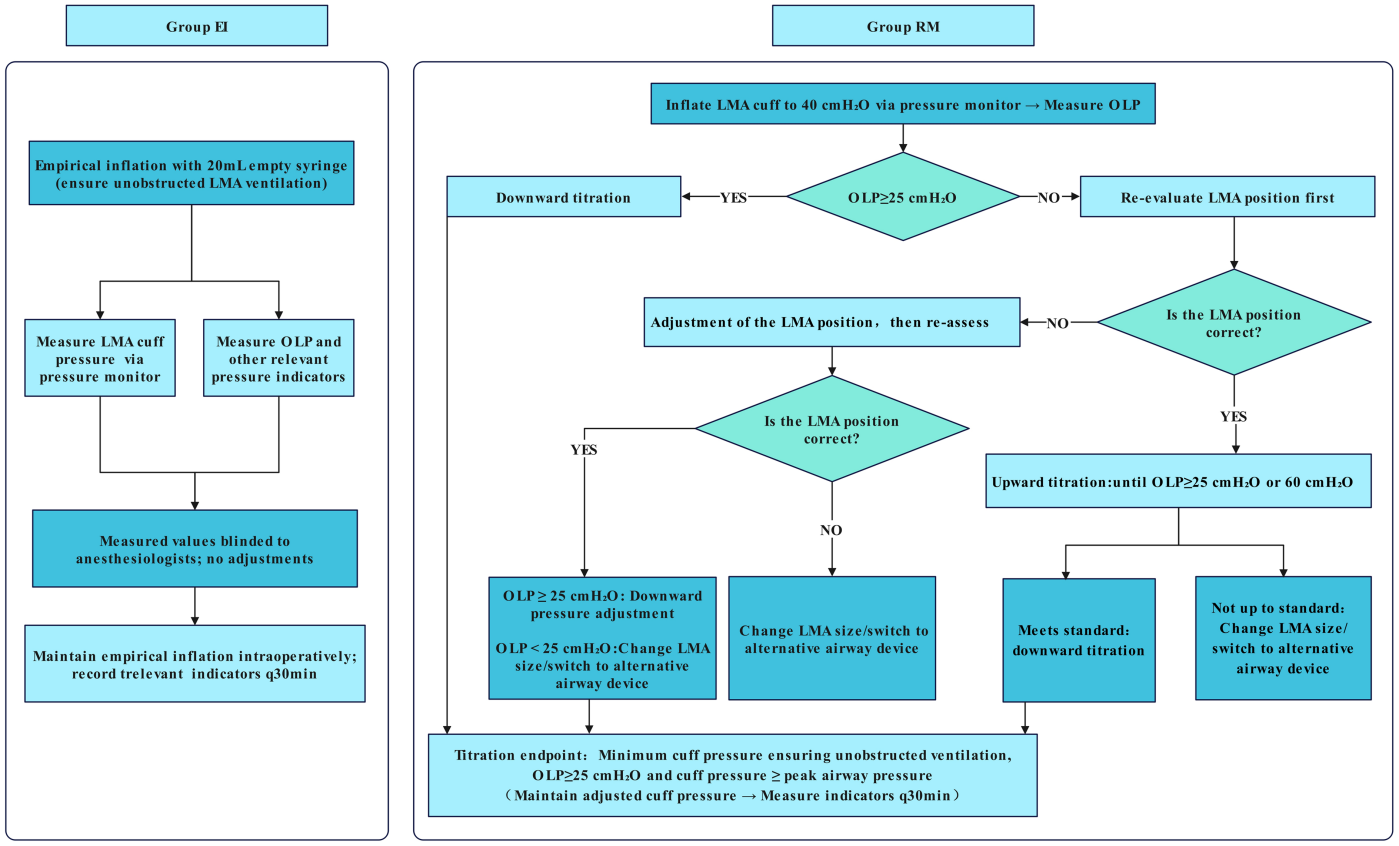
Schematic diagram of experimental grouping procedure.

Individualized regulation proceeded as follows:

1. If OLP was ≥ 25 cmH_2_O: pressure was reduced in 2 cmH_2_O increments to the minimum value maintaining unobstructed ventilation, OLP ≥ 25 cmH_2_O, and cuff pressure ≥ peak airway pressure.
2. If the OLP < 25 cmH₂O: ILMA position was rechecked; with correct placement, pressure was increased in 5 cmH₂O increments until OLP ≥ 25 cmH₂O or 60 cmH₂O (safety limit). If OLP met the target after upward adjustment, downward regulation was performed as above. For regulation failure (OLP < 25 cmH₂O at 60 cmH₂O) or persistent ILMA malposition, ILMA size was changed, or alternative supraglottic airway device/endotracheal intubation was used to ensure patient safety (Figure 1). This target cuff pressure was maintained continuously using the pressure monitor throughout the procedure. Indicators including tidal volume, airway pressure, and OLP were recorded every 30 min, and the average values were calculated for analysis.

## 3 Outcomes

### 3.1 Primary outcome

The primary outcome measure was the incidence of pharyngolaryngeal pain within 48 h after ILMA removal in participants. Assessment time points included immediately after ILMA removal (T1), 10 min (T2), 30 min (T3), 1 h (T4), 2 h (T5), 24 h (T6), and 48 h (T7) post removal. The Visual Analogue Scale (VAS) for pain (13) was used, with a score of ≥3 defined as the presence of pharyngolaryngeal pain.

### 3.2 Secondary outcomes

The secondary outcome measures included the incidence of supraglottic pharyngeal mucosal injury, bloodstains on the surface of the ILMA or in sputum, hoarseness and swallowing discomfort; the difficulty level, number of attempts and time duration of ILMA insertion (14); bronchoscopic ILMA positional grading (15, 16); ILMA OLP; other perioperative airway complications; the incidence of postoperative pulmonary complications within 7 days (17); as well as the length of hospital stay, hospitalization costs and patient satisfaction.

Bronchoscopic ILMA position was graded on a 4-point scale according to glottic visibility. Supraglottic mucosal injury was assessed using a predefined bronchoscopy-based grading scale, and was defined as an increase in grade from before ILMA insertion to after ILMA removal. Visible blood staining on the ILMA was defined as any visible fresh blood or blood staining on the cuff or shaft immediately after removal. Hoarseness was assessed using a 4-point scale, and patient satisfaction was evaluated before discharge using a 3-item 5-point Likert scale. Detailed definitions and assessment procedures for selected secondary outcomes are provided in the Supplementary material.

### 3.3 Statistical analysis

The primary outcome measure is the incidence of pharyngolaryngeal pain within 48 h after removal of the ILMA. Based on preliminary pilot study results, the expected incidence of pharyngolaryngeal pain was 10% in group RM and 41.67% in group EI. With a two-sided *α* level of 0.05 and a power of 90%, the sample size was calculated using PASS 15.0 software, yielding a sample size of 35 cases in group RM and 35 cases in group EI. Considering a 10% rate of loss to follow-up and refusal, a minimum of 39 participants were required for each group, with a total of at least 78 participants to be enrolled in the study.

The data analysis of this study was performed based on the ITT principle. All patients enrolled and randomized (*N* = 78) were included in the analysis of primary outcome measures. In addition, a per-protocol (PP) analysis was concurrently conducted in this study (*N* = 74) to verify the robustness of the results. For statistical analysis, the normality of continuous variables was assessed using the Shapiro–Wilk test. Variables conforming to a normal distribution were described as mean±standard deviation (SD) and compared between groups using the independent-samples *t*-test. For non-normally distributed variables, median (interquartile range, IQR) was used for description, and the Mann–Whitney U test was applied for intergroup comparisons. Categorical data were presented as number of cases (percentage) and analyzed with the chi-square test or Fisher’s exact test for between-group comparisons. To further investigate the primary outcome measures, this study employed a multivariate logistic regression model to adjust for potential confounding factors. All data processing and analyses were performed using SPSS 25.0 statistical software. A *p* < 0.05 was considered statistically significant. For the time-point-specific comparisons of postoperative pharyngolaryngeal pain incidence across T1–T7, *p* values were adjusted for multiple comparisons using the Holm method.

## 4 Results

### 4.1 Participant flow and baseline characteristics

**Figure 2:**
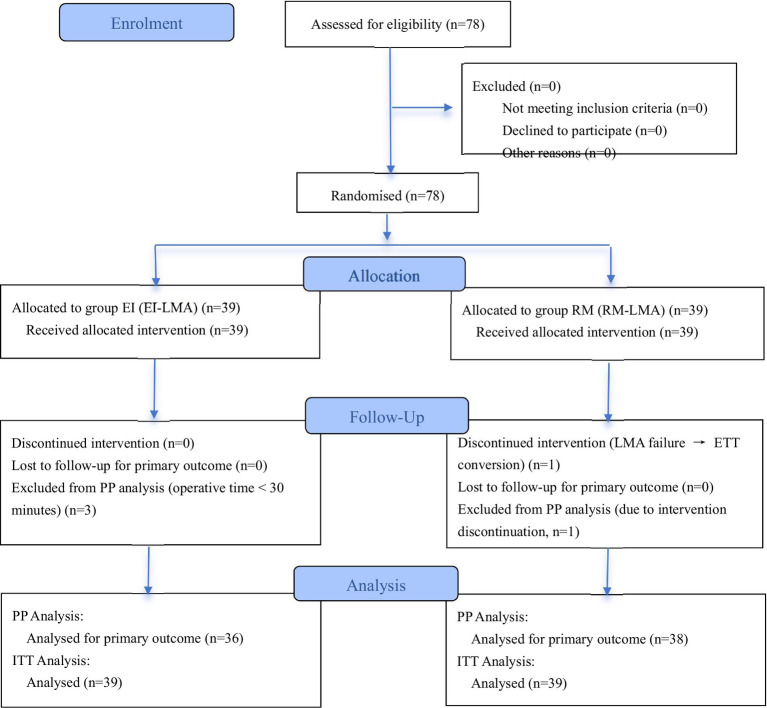
CONSORT flow chart describing participant flow.

A total of 78 participants were enrolled and randomly assigned to the group EI and group RM, with 39 participants in each group. According to the per-protocol analysis principle, 3 participants in the group EI (due to surgery duration <30 min) and 1 participant in the group RM (due to switching to endotracheal intubation ventilation because of poor ILMA ventilation efficacy) were excluded. Ultimately, 74 participants completed the study (Figure 2). The ITT analysis and PP analysis both demonstrated balanced baseline demographic and clinical characteristics between groups, with no statistically significant differences (*p* > 0.05), indicating comparability (Table 1; Supplementary Table 1).

**Table 1:** Baseline characteristics of the patients (ITT analysis).

Variables	All patients	EI-LMA	RM-LMA	*P* value
	*N* = 78	*N* = 39	*N* = 39	
Sex, female%	32 (41.0%)	13 (33.3%)	19 (48.7%)	0.170
Age, years	70 (65, 75)	71 ± 7	70 ± 8	0.671
Height, cm	161.8 ± 8.7	163.1 ± 7.8	160 (150, 170)	0.290
Weight, kg	60.6 ± 8.7	62.5 ± 9.3	58.7 ± 7.8	0.056
BMI, kg/m^2^	23.1 ± 2.5	23.4 ± 2.5	22.8 ± 2.5	0.277
IID, cm	4.3 (4.2, 4.5)	4.5 (4.2, 4.6)	4.2 (4.1, 4.5)	0.092
TMD, cm	6.2 (6.0, 6.4)	6.3 ± 0.3	6.1 ± 0.4	0.108
NYHA classification				0.501
I	10 (12.8%)	4 (10.3%)	6 (15.4%)	
II	68 (87.2%)	35 (89.7%)	33 (84.6%)	
ASA physical status				0.260
II	37 (47.4%)	16 (41.0%)	21 (53.8%)	
III	41 (52.6%)	23 (59.0%)	18 (46.2%)	
Mallampati classification				0.194
I	13 (16.7%)	4 (10.3%)	9 (23.1%)	
II	61 (78.2%)	33 (84.6%)	28 (71.8%)	
III	3 (3.8%)	1 (2.6%)	2 (5.1%)	
IV	1 (1.3%)	1 (2.6%)	0	
Comorbidities				0.650
Hypertension, *n*%	31 (39.7%)	18 (46.2%)	13 (33.3%)	
Diabetes, *n*%	13 (16.7%)	6 (15.4%)	7 (17.9%)	
Coronary heart disease, *n*%	4 (5.1%)	2 (5.1%)	2 (5.1%)	
Cerebrovascular disease, *n*%	5 (6.4%)	3 (7.7%)	2 (5.1%)	
Arrhythmology, *n*%	4 (5.1%)	2 (5.1%)	2 (5.1%)	
ARISCAT score				0.924
3	66 (84.6%)	33 (84.6%)	33 (84.6%)	
11	1 (1.3%)	1 (2.6%)	0	
16	8 (10.3%)	4 (10.3%)	4 (10.3%)	
24	1 (1.3%)	1 (2.6%)	0	
27	1 (1.3%)	0	1 (2.6%)	
40	1 (1.3%)	0	1 (2.6%)	
FRAIL score				0.254
0	64 (82.1%)	34 (87.2%)	30 (76.9%)	
1	12 (15.4%)	4 (10.3%)	8 (20.5%)	
2	2 (2.6%)	1 (2.6%)	1 (2.6%)	
Preoperative clinical parameters				
Systolic pressure, mmHg	135 ± 17	138 ± 14	133 ± 19	0.235
Dyastolic pressure, mmHg	79 ± 12	80 ± 11	78 ± 12	0.374
Heart rate, bpm	78 (73, 86)	78 (72, 86)	77 (73, 86)	0.787
Ventilatory frequency, bpm	19 (17, 20)	20 (16, 20)	18 (17, 20)	0.280
SpO_2_ in room air	98 (97, 99)	98 (97, 99)	98 (97, 99)	0.146
Preoperative electrocardiogram, abnormal %	44 (56.4%)	23 (59.0%)	21 (53.8%)	0.650
Surgery type				0.122
Urological, *n*%	58 (74.4%)	32 (82.1%)	26 (66.7%)	
Osteoarticular, *n*%	20 (25.6%)	7 (17.9%)	13 (33.3%)	

Values are mean ± SD, median [IQR] or *n* (%). BMI, body mass index; NYHA classification, New York Heart association classification; ASA physical status, American Society of Anesthesiologists Physical Status Classification System; IID, inter-incisal distance; TMD, thyromental distance; ARISCAT, assess respiratory risk in surgical patients in Catalonia.

### 4.2 Intraoperative parameters and operational performance

In both the ITT and PP analyses, compared with group EI, group RM achieved significantly lower ILMA cuff pressure values through individualized regulation (all *P*<0.05). No statistically significant between-group differences were observed in ILMA insertion time, ILMA size, insertion difficulty grade, number of insertion attempts, bronchoscopic LMA positional grade, OLP, airway pressure, other perioperative airway complications, duration of surgery, or opioid consumption (all *p* > 0.05). Moreover, when the initial cuff pressure in group RM was set at 40 cmH₂O, all OLP values were ≥25 cmH₂O. This indicates that continuous monitoring and individualized regulation of ILMA cuff pressure can effectively achieve pressure control targets without compromising ILMA performance or intraoperative anesthesia management (Table 2; Supplementary Table 2).

**Table 2:** Intraoperative characteristics of the patients (ITT analysis).

Variables	All patients	EI-LMA	RM-LMA	*P* value
	*N* = 78	*N* = 39	*N* = 39	
Time for ILMA insertion, s	8 (7, 9)	8 (7, 9)	8 (7, 9)	0.560
ILMA size (#)				0.258
3.0	35 (44.9%)	15 (38.5%)	20 (51.3%)	
4.0	43 (55.1%)	24 (61.5%)	19 (48.7%)	
Difficulty grade of ILMA insertion				0.316
1	67 (85.9%)	32 (82.1%)	35 (89.7%)	
2	10 (12.8%)	6 (15.4%)	4 (10.3%)	
3	1 (1.3%)	1 (2.6%)	0	
Number of ILMA insertion attempts				0.079
1	75 (96.2%)	39 (100%)	36 (92.3%)	
2	3 (3.8%)	0	3 (7.7%)	
Bronchoscopic position grade				0.675
4	59 (75.3%)	29 (74.4%)	30 (76.9%)	
3	12 (15.6%)	5 (12.8%)	7 (17.9%)	
2	3 (3.9%)	1 (2.6%)	2 (5.1%)	
1	4 (5.2%)	4 (10.3%)	0	
ILMA CP*	**30 (25, 51)**	**50 ± 21**	**26 (23, 28)**	**<0.001**
ILMA OLP	28 (26, 29)	27 (26, 29)	28 (27, 29)	0.093
Airway pressure	14 (13, 15)	14 (13, 15)	13 (13, 15)	0.360
Other perioperative airway complications, positive %	5 (6.4%)	3 (7.7%)	2 (5.1%)	0.646
Duration of the surgery, min	42 (30, 59)	45 (30, 56)	42 (30, 65)	0.519
Dosage of sufentanil, μg	25 (20, 30)	20 (20, 30)	25 (20, 30)	0.150
Dosage of remifentanil, mg	0.37 (0.26, 0.59)	0.34 (0.26, 0.53)	0.43 (0.26, 0.67)	0.296

ILMA, Inflatable Laryngeal Mask Airway; ILMA CP, ILMA cuff pressure; ILMA OLP, ILMA oropharyngeal leakage pressure; Bold values indicate statistically significant differences between groups (p < 0.05). *: *p* < 0.05.

### 4.3 Postoperative outcomes and complications

Intergroup comparisons of postoperative complications were performed using Fisher’s exact test. In the ITT analysis, the incidence of pharyngolaryngeal pain within 48 h after ILMA removal [7.7% (3/39) vs. 35.9% (14/39), *p* = 0.003] and the incidence of supraglottic mucosal injury [0% (0/39) vs. 15.4% (6/39), *p* = 0.011] were significantly lower in the group RM than in the group EI (Table 3). Similar findings were observed in the PP analysis, in which the incidence of pharyngolaryngeal pain [7.9% (3/38) vs. 36.1% (13/36), *p* = 0.002] and the incidence of supraglottic mucosal injury [0% (0/38) vs. 16.7% (6/36), *p* = 0.007] also significantly lower in the group RM than in the group EI (Supplementary Table 3). No statistically significant between-group differences were observed in visible bloodstaining on the ILMA, blood-tinged sputum, hoarseness, dysphagia, postoperative pulmonary complications within 7 days, analgesic pump use, length of hospital stay, total hospitalization costs, or patient satisfaction (all *p* > 0.05).

**Table 3:** Postoperative characteristics of the patients (ITT analysis).

Variables	All patients	EI-LMA	RM-LMA	*P* value
	*N* = 78	*N* = 39	*N* = 39	
Duration of ILMA placement (min)	81 ± 30	79 ± 27	82 ± 33	0.812
Visible blood staining on the ILMA (positive%)	3 (3.8%)	2 (5.1%)	1 (2.6%)	0.559
Pharyngolaryngeal pain within 48 h (positive%)*	**17 (21.8%)**	**14 (35.9%)**	**3 (7.7%)**	**0.003**
Hoarseness (positive%)	14 (17.9%)	8 (20.5%)	6 (15.4%)	0.558
Dysphagia (positive%)	3 (3.8%)	2 (5.1%)	1 (2.6%)	0.559
Blood-tinged sputum (positive%)	2 (2.6%)	2 (5.1%)	0	0.155
Laryngeal mucosa injury above the glottis (positive%)*	**6 (7.7%)**	**6 (15.4%)**	**0**	**0.011**
7-day pulmonary complications (positive%)	1 (1.3%)	1 (2.6%)	0	0.317
Use of analgesic pump (yes, %)	18 (23.1%)	7 (17.9%)	11 (28.2%)	0.285
Length of hospital stay (day)	6 (5, 7)	6 (5, 7)	7 (5, 8)	0.712
Total hospitalization Cost (w)	1.4 (1.2, 1.9)	1.4 (1.1, 1.9)	1.5 (1.2, 2.5)	0.325
Patient satisfaction (point)	15 (14, 15)	15 (14, 15)	15 (14, 15)	0.751

Bold values indicate statistically significant differences between groups (*p* < 0.05).*: *p* < 0.05.

**Figure 3:**
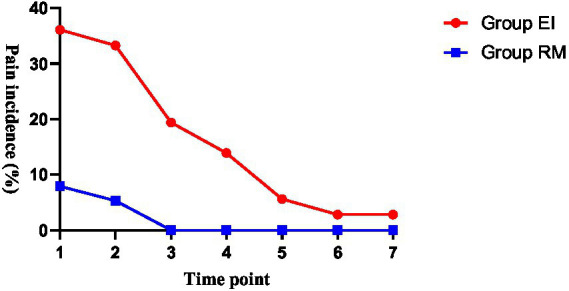
Temporal changes in postoperative pharyngolaryngeal pain incidence in the EI and RM groups. Pain incidence at each time point was defined as the proportion of participants with a VAS score ≥ 3. Time points 1–7 correspond to T1–T7, representing immediately after ILMA removal, 10 min, 30 min, 1 h, 2 h, 24 h, and 48 h, respectively.

**Figure 4:**
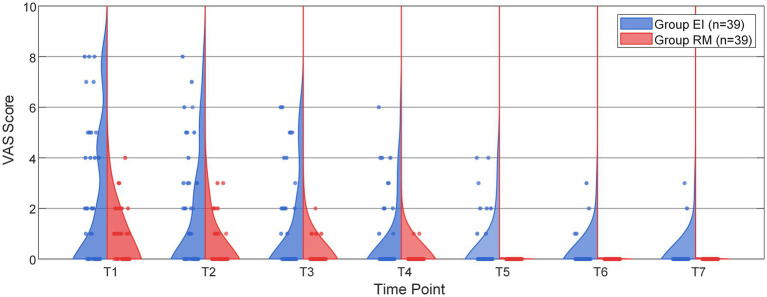
Distribution of postoperative pain VAS scores over time in the EI and RM groups. The violin plots display the distribution of VAS scores for postoperative pharyngolaryngeal pain at T1–T7. T1–T7 correspond to 0 min, 10 min, 30 min, 1 h, 2 h, 24 h, and 48 h after ILMA removal, respectively.

To further clarify the temporal pattern of postoperative pain, time-point-specific analyses of pain incidence were performed. In the ITT population, the incidence of postoperative pharyngolaryngeal pain was significantly lower in the RM group than in the EI group at T1, T2, and T3 after Holm correction for multiple comparisons, whereas no statistically significant between-group differences were observed at T4–T7 (Figure 3; Table 4). Similar temporal patterns were observed in the PP population, in which the between-group differences also remained statistically significant at T1, T2, and T3 after Holm correction, but not at T4–T7 (Supplementary Table 4). The violin plots also illustrated a similar temporal pattern in VAS score distribution, with lower scores in the RM group during the early postoperative period (Figure 4).

**Table 4:** Time-point-specific comparison of postoperative pharyngolaryngeal pain incidence (ITT analysis).

Time point	All patients	EI-LMA	RM-LMA	*P* value	Holm-adjusted *P*
	*N* = 78	*N* = 39	*N* = 39		
T1*	**17 (21.8%)**	**14 (35.9%)**	**3 (7.7%)**	**0.003**	**0.018**
T2*	**15 (19.2%)**	**13 (33.3%)**	**2 (5.1%)**	**0.002**	**0.014**
T3*	**8 (10.3%)**	**8 (20.5%)**	**0**	**0.005**	**0.025**
T4	6 (7.7%)	6 (15.4%)	0	0.025	0.100
T5	3 (3.8%)	3 (7.7%)	0	0.240	0.720
T6	1 (1.3%)	1 (2.6%)	0	1.000	1.000
T7	1 (1.3%)	1 (2.6%)	0	1.000	1.000

Pain incidence was defined as a VAS score ≥3. T1-T7 correspond to 0 min, 10 min, 30 min, 1 h, 2 h, 24 h, and 48 h after ILMA removal, respectively. *P* values were adjusted for multiple comparisons using the Holm method. Bold values indicate statistically significant differences between groups (*p* < 0.05).*: *p* < 0.05.

### 4.4 Multivariate regression

In the ITT analysis, multivariate logistic regression analysis confirmed that cuff pressure was an independent risk factor for pharyngolaryngeal pain after adjusting for surgery duration, cuff retention time, and OLP (adjusted odds ratio = 1.037, 95% confidence interval: 1.008–1.067, *p* = 0.011).

## 5 Discussion

This prospective RCT specifically explored the effect of individualized regulation of ILMA cuff pressure on postoperative pharyngolaryngeal complications in elderly patients. Compared with routine empirical inflation, individualized regulation to maintain lower cuff pressures significantly reduced the incidence of postoperative pharyngolaryngeal complications without compromising ventilation seal integrity or anesthetic procedure safety. Multivariate regression analysis further confirmed that cuff pressure is an independent risk factor for postoperative pharyngolaryngeal pain. These findings indicate that optimizing and maintaining cuff pressure at an appropriate level represents a key and feasible anesthetic management strategy to enhance perioperative patient comfort and promote accelerated postoperative recovery.

An important finding was that the protective effect of individualized cuff pressure regulation was mainly confined to the early postoperative period. In both the ITT and PP analyses, after Holm correction for multiple comparisons, the between-group difference in pain incidence remained significant at T1, T2, and T3, but not at later time points. This temporal pattern is clinically plausible, because excessive cuff pressure is more likely to cause early mucosal compression and transient local ischemic irritation, with symptoms most evident immediately after ILMA removal and during early recovery. As local tissue irritation gradually resolves, the between-group difference may diminish over time. These findings suggest that the principal benefit of individualized cuff pressure regulation lies in alleviating early postoperative discomfort.

Importantly, the reduction in postoperative pharyngolaryngeal pain was achieved without compromising ventilation seal integrity. In both the ITT and PP analyses, OLP did not differ significantly between groups, suggesting that individualized cuff pressure regulation can improve postoperative comfort while maintaining adequate airway sealing. This finding supports the clinical feasibility of pressure titration to the lowest effective level in elderly patients.

The airway management mechanism of ILMAs relies on anatomical alignment sealing and mechanical pressure sealing formed by the cuff in the pharynx (13). Previous studies suggested that post-ILMA pharyngolaryngeal discomfort correlates with mechanical ventilation type rather than cuff pressure variations (18). This discrepancy likely stems from differences in study populations and pressure management strategies. Our study focused on elderly patients, and the implementation of active pressure control underscores the critical importance of maintaining cuff pressure within safe limits for this demographic. Additional studies support that appropriately reducing cuff pressure does not affect OLP while reducing postoperative dysphagia incidence (19). Notably, a previous study involving relatively younger patients (mean age ~50 years) using LMA Supreme™ suggested that increasing cuff pressure to 80 cmH_2_O significantly elevated OLP without increasing postoperative laryngeal complications (20). This difference may be related to variations in patient age, airway device material, and study objectives. That study aimed to identify higher effective seal pressures for a specific LMA, whereas our study focused on determining the lowest cuff pressure required to maintain adequate sealing.

The stepwise regulation protocol employed in this study is particularly physiologically rational for the elderly. Given the reduced mucosal compliance and impaired microcirculatory reserve in this population, a gradual individualized pressure adjustment allows identification of the precise threshold that maintains adequate sealing without inducing mucosal ischemia. This approach is inherently safer than targeting a generic “low” pressure bracket (e.g., <60 cmH₂O), as it accounts for individual anatomical variability and dynamically adapts to the patient’s real-time airway pressure. The operationalization of this protocol was guided by two physiologically grounded safety criteria: first, the titrated cuff pressure must be sufficient to counteract the patient’s peak airway pressure, ensuring baseline seal integrity; second, to provide a consistent safety margin accounting for dynamic intraoperative conditions, the measured OLP was maintained at ≥ 25 cmH₂O. This dual-threshold approach—coupling an individualized lower limit (against airway pressure) with a general safety floor (25 cmH₂O OLP)—ensured that the quest for the “lowest effective pressure” never compromised ventilatory safety, particularly in this vulnerable cohort.

The study has several limitations. First, although the sample size met the prespecified requirement for the primary outcome, this was still a single-center study with a relatively limited sample size. As a result, the study was not sufficiently powered for robust subgroup analyses by sex or age, and these findings should therefore be further validated in larger multicenter studies. Second, because the intervention required real-time cuff pressure monitoring and active pressure adjustment, blinding of the anesthesiologists performing ILMA insertion and cuff management was not feasible, which may have introduced performance bias. However, patients, postoperative outcome assessors, and data analysts remained blinded to group allocation. Third, the regulation parameters used in this study were selected based on theory, pilot observations, and clinical practice. Although the regulation algorithm was standardized, it involved a fixed decrement of 2 cmH_2_O for downward adjustment and an increment of 5 cmH_2_O for upward adjustment. Future studies may further optimize the individualized regulation protocol by systematically comparing different initial pressures and adjustment step sizes.

## 6 Conclusion

To our knowledge, this is among the first randomized studies to specifically evaluate individualized ILMA cuff pressure regulation in an elderly population. Our findings suggest that continuous monitoring and individualized regulation of ILMA cuff pressure to the minimum effective pressure that maintains OLP ≥ 25 cmH_2_O while exceeding peak airway pressure may reduce the incidence of postoperative pharyngolaryngeal pain and other complications in elderly patients, particularly during the early postoperative period. This approach may represent a feasible and clinically relevant anesthetic strategy for improving perioperative comfort and supporting ERAS in this population.

## Data Availability

The raw data supporting the conclusions of this article will be made available by the authors, without undue reservation.
