# Supplementary material for: Individualized regulation of inflatable laryngeal mask airway cuff pressure reduces postoperative pharyngolaryngeal pain in elderly patients: a randomized controlled trial
Source: Front Med (Lausanne). 2026 May 15;13:1803147. doi: 10.3389/fmed.2026.1803147 (PMC13219002; doi:10.3389/fmed.2026.1803147)
Supplement: Supplementary file 1 [file Data_Sheet_1.PDF]

Supplementary Material

1 Supplementary Tables and Methods

1.1 Supplementary Table 1

Baseline characteristics of the patients. (PP Analysis)

Variables	All patients N=74	EI-LMA N=36	RM-LMA N=38	*P* value
Sex, female%	31(41.9%)	12(33.3%)	19(50.0%)	0.149
Age, years	70(65,74)	71±7	69(63,74)	0.413
Height, cm	161.8±8.9	163±8.1	160(150,170)	0.115
Weight, kg	60.6±8.9	62.4±9.6	58.8±7.9	0.086
BMI, kg/m^2	23.1±2.5	23.3±2.6	22.9±2.4	0.460
IID, cm	4.3(4.2,4.5)	4.5(4.2,4.5)	4.3(4.2,4.5)	0.122
TMD, cm	6.2(6.0,6.4)	6.2(6.0,6.4)	6.1(5.9,6.4)	0.092
NYHA classification				0.559
I	10(13.5%)	4(11.1%)	6(15.8%)	
II	64(86.5%)	32(88.9%)	32(84.2%)	
ASA physical status				0.161
II	35(47.3%)	14(38.9%)	21(55.3%)	
III	39(52.7%)	22(61.1%)	17(44.7%)	
Mallampati Classification				0.215
I	13(17.6%)	4(11.1%)	9(23.7%)	
II	57(77.0%)	30(83.3%)	27(71.1%)	
III	3(4.1%)	1(2.8%)	2(5.3%)	
IV	1(1.4%)	1(2.8%)	0	

Comorbidities				0.840
Hypertension, n%	29(39.2%)	17(47.2%)	12(31.6%)	
Diabetes, n%	11(14.9%)	5(13.9%)	6(15.8%)	
Coronary heart disease, n%	3(4.1%)	2(5.6%)	1(2.6%)	
Cerebrovascular disease, n%	4(5.4%)	2(5.6%)	2(5.3%)	
Arrhythmology, n%	3(4.1%)	2(5.6%)	1(2.6%)	
ARISCAT score				1.000
3	62(83.8%)	30(83.3%)	32(84.2%)	
11	1(1.4%)	1(2.8%)	0	
16	8(10.8%)	4(11.1%)	4(10.5%)	
24	1(1.4%)	1(2.8%)	0	
27	1(1.4%)	0	1(2.6%)	
40	1(1.4%)	0	1(2.6%)	
FRAIL score				0.265
0	62(83.8%)	32(88.9%)	30(78.9%)	
1	10(13.5%)	3(8.3%)	7(18.4%)	
2	2(2.7%)	1(2.8%)	1(2.6%)	
Preoperative clinical parameters				
Systolic pressure, mmHg	135(126,146)	137±14	134±19	0.324
Dyastolic pressure, mmHg	80(72,86)	80±11	78±12	0.606
Heart rate, bpm	78(72,86)	78(71,86)	77(73,86)	0.927

Ventilatory frequency, bpm	19(16,20)	20(16,20)	18(17,20)	0.360
SpO_2 in room air	98(97,99)	98(97,99)	99(97,99)	0.104
Preoperative electrocardiogram, abnormal%	41(55.4%)	21(58.3%)	20(52.6%)	0.624
Surgery type				0.236
Urological, n%	55(74.3%)	29(80.6%)	26(68.4%)	
Osteoarticular, n%	19(25.7%)	7(19.4%)	12(31.6%)	

Values are mean ± SD, median [IQR] or n (%).

BMI: Body mass index, NYHA classification: New York Heart Association classification, ASA physical status: American Society of Anesthesiologists Physical Status Classification System, IID:Inter-incisal Distance, TMD:Thyromental Distance, ARISCAT:Assess Respiratory Risk in Surgical Patients in Catalonia.

1.2 Supplementary Table 2

Intraoperative characteristics of the patients. (PP Analysis)

**Variables**	**All patients N=74**	**EI-LMA N=36**	**RM-LMA N=38**	***P* value**
Time for LMA insertion, s	8(7,9)	8(7,9)	8(6,9)	0.377
ILMA size（#）				0.239
3.0	34(45.9%)	14(38.9%)	20(52.6%)	
4.0	40(54.1%)	22(61.1%)	18(47.4%)	
Difficulty grade of ILMA insertion				0.269
1	63(85.1%)	29(80.6%)	34(89.5%)	

2	10(13.5%)	6(16.7%)	4(10.5%)	
3	1(1.4%)	1(2.8%)	0	
Number of ILMA insertion attempts				0.087
1	71(95.9%)	36(100%)	35(92.1%)	
2	3(4.1%)	0	3(7.9%)	
Bronchoscopic position grade				0.762
4	56(75.7%)	27(75%)	29(76.3%)	
3	12(16.2%)	5(13.9%)	7(18.4%)	
2	3(4.1%)	1(2.8%)	2(5.3%)	
1	3(4.1%)	3(8.3%)	0	
**ILMA CP ***	**28(25,51)**	**51(38,65)**	**26(22,27)**	**P＜0.001**
ILMA OLP	28(26,29)	27(26,29)	28(27,30)	0.316
Airway Pressure	14(12,15)	14(12,15)	13(12,15)	0.402
Other perioperative airway complications, positive %	4(5.4%)	2(5.6%)	2(5.3%)	0.912
Duration of the surgery, min	45(30,59)	45(30,59)	41(30,64)	0.939
Dosage of sufentanil, μg	25(20,30)	20(20,30)	25(20,30)	0.198
Dosage of remifentanil, mg	0.38(0.26,0.59)	0.40±0.16	0.44±0.22	0.689

ILMA OLP: Laryngeal mask airway oropharyngeal leakage pressure, ILMA CP: Laryngeal mask airway cuff pressure. *: $P < 0.05$.

1.3 Supplementary Table 3

Postoperative characteristics of the patients. (PP Analysis)

Variables	All patients N=74	EI-LMA N=36	RM-LMA N=38	*P* value
Duration of ILMA placement (min)	78(58,105)	80(59,99)	74(55,106)	0.948
Visible blood staining on the ILMA, (positive%)	3(4.1%)	2(5.6%)	1(2.6%)	0.610
**Pharyngolaryngeal pain within 48h (positive%) ***	**16(21.6%)**	**13(36.1%)**	**3(7.9%)**	**0.002**
Hoarseness (positive%)	14(18.9%)	8(22.2%)	6(15.8%)	0.261
Dysphagia (positive%)	3(4.1%)	2(5.6%)	1(2.6%)	0.610
Blood-tinged sputum (positive%)	2(2.7%)	2(5.6%)	0	0.233
**Laryngeal mucosa injury above the glottis, positive % ***	**6(8.1%)**	**6(16.7%)**	**0**	**0.007**
7-day pulmonary complications (positive%)	1(1.4%)	1(2.8%)	0	0.486
Use of analgesic pump (yes, %)	18(24.3%)	7(19.4%)	11(28.9%)	0.341
Length of Hospital Stay	6(5,7)	6(5,8)	7(5,7)	0.991
Total Hospitalization Cost(w)	1.5(1.2,1.9)	1.5(1.2,1.9)	1.4(1.2,2.4)	0.611
Patient Satisfaction	15(14,15)	15(15,15)	15(14,15)	0.476

*: $P < 0.05$.

1.4 Supplementary Table 4

Time-point-specific comparison of postoperative pharyngolaryngeal pain incidence in the PP population. (PP Analysis)

Time point	All patients N=74	EI-LMA N=36	RM-LMA N=38	*P* value	Holm-adjusted *P*
**T1 ***	**16(21.6%)**	**13(36.1%)**	**3(7.9%)**	**0.002**	**0.012**
**T2 ***	**14（18.9%）**	**12（33.3%）**	**2（5.3%）**	**0.001**	**0.007**
**T3 ***	**7（9.5%）**	**7（19.4%）**	**0**	**0.004**	**0.020**
T4	5（6.8%）	5（13.9%）	0	0.020	0.080
T5	2（2.7%）	2（5.6%）	0	0.220	0.660
T6	1（1.4%）	1（2.8%）	0	0.473	0.946
T7	1（1.4%）	1（2.8%）	0	0.473	0.946

Pain incidence was defined as a VAS score ≥ 3. T1–T7 correspond to 0 min, 10 min, 30 min, 1 h, 2 h, 24 h, and 48 h after ILMA removal, respectively. *P* values were adjusted for multiple comparisons using the Holm method. *: $P < 0.05$.

1.5 Supplementary Methods

Detailed definitions and assessment procedures for secondary outcomes

Bronchoscopic ILMA positional grade was assessed under fiberoptic bronchoscopy after ILMA placement. The grading criteria were as follows: grade 4, full view of the glottis; grade 3, partial view of the glottis; grade 2, no direct view of the glottis but visible after adjustment; and grade 1, glottis not identifiable. With patients in the supine position (neutral head position) under adequate anesthetic depth, a V-BFCP-450 video bronchoscope (Weisibo Medical Technology Co., Ltd., Hangzhou, China) was used to examine and record supraglottic mucosal condition pre-insertion, assess ILMA position post-insertion, and conduct a repeat assessment in the RM group after cuff pressure adjustment.

Supraglottic mucosal injury was evaluated under fiberoptic bronchoscopy before ILMA insertion and again after ILMA removal using a predefined 4-grade scale: grade 0, no hemorrhage; grade 1, one hemorrhagic spot; grade 2, two hemorrhagic spots; and grade 3, three or more hemorrhagic spots or

visible fresh blood. Mucosal injury was defined as an increase in grade from pre-insertion to post-removal assessment.

Visible blood staining on the ILMA was recorded as a binary outcome immediately after device removal. A positive result was defined as the presence of any visible fresh blood or blood staining on the cuff or shaft of the ILMA. This assessment was performed by an independent assessor blinded to group allocation.

Hoarseness was evaluated postoperatively using a 4-point scale: grade 0, no hoarseness; grade 1, slight change in voice quality reported only on questioning; grade 2, obvious change in voice quality reported on questioning; and grade 3, obvious change in voice quality spontaneously noticed by the patient and family members.

Difficulty of ILMA insertion was graded as follows: grade 1, minimal resistance with satisfactory ventilation achieved without adjustment; grade 2, moderate resistance requiring auxiliary maneuvers such as the up-down maneuver, assistant jaw support, or adjustment of cuff inflation volume; and grade 3, marked resistance requiring reinsertion, device size replacement, or reverse insertion technique.

Patient satisfaction was assessed before discharge using a 3-item 5-point Likert scale covering perioperative comfort, postoperative recovery, and investigator attitude. Each item was scored as 1 = very dissatisfied, 2 = dissatisfied, 3 = neutral, 4 = satisfied, and 5 = very satisfied, giving a total score range of 3-15, with higher scores indicating greater satisfaction.
